# Editorial: Algorithm-hardware co-optimization in neuromorphic computing for efficient AI

**DOI:** 10.3389/fnins.2025.1746610

**Published:** 2025-12-04

**Authors:** Amirreza Yousefzadeh, Alberto Patiño-Saucedo, Guido De Croon, Manolis Sifalakis

**Affiliations:** 1 CAES, University of Twente, Enschede, Netherlands; 2 IMSE-CNM, CSIC, Seville, Spain; 3 MAVL, TU-Delft, Delft, Netherlands; 4 Innatera, Delft, Netherlands

**Keywords:** neuromorphic computing, algorithm-hardware co-optimization, spiking neural networks (SNNs), temporal efficiency/timestep reduction, hardware-aware mapping and learning

## 1 Introduction

Neuromorphic computing holds the promise of sustainable AI by combining brain-inspired models with event-driven, massively parallel hardware. However, a central question remains: when and how do neuromorphic systems convert their architectural advantages into effective end-to-end efficiency for real-world tasks? This Research Topic presents six contributions that address this question from various perspectives. Specifically, it explores training methods that minimize timesteps and memory usage, hardware-aware algorithms and quantization, emulation techniques that mitigate risks associated with analog platforms, and mapping and scheduling strategies that enhance utilization on many-core neuromorphic chips.

## 2 About this Research Topic

We are pleased to present a series of innovative research articles in this field that introduce the following techniques:

- Early-exit and regularization for fewer timesteps: Wu, Dengyu, et al. address a simple but important inefficiency in spiking neural networks (SNNs): many inputs do not need the full simulation horizon to be classified correctly. They introduce a general “cutoff" strategy that encourages the network to stop early when it is already confident. Concretely, they use a Top-K cutoff, meaning the model monitors the top-scoring classes during inference and terminates once the top candidates remain stable. A matching training regularizer teaches the SNN to reach reliable decisions sooner. Across both frame-based and event-based datasets, this leads to substantially fewer timesteps with negligible accuracy loss, and the method applies to both ANN-to-SNN conversion and direct SNN training.
- Integer SNNs with dynamic thresholds for hardware affinity: Zou et al. propose an all-integer Spiking Neural Network (SNN) with a dynamic threshold adaptation that eliminates synchronization errors typically encountered in the conversion from Artificial Neural Networks (ANN) to SNN.
- Learning methods that do not rely on gradients and are compatible with the constraints of neuromorphic systems: Fernández et al. investigate perturbation-based learning for recurrent neural networks. They improve upon the existing method of activity-based node perturbation by applying it to the time domain and introducing a decorrelation mechanism. Their approach competes with Backpropagation Through Time (BPTT) in terms of performance and convergence, while also maintaining a purely forward-pass training process. Furthermore, it utilizes a simple global reinforcement signal, making it an appealing option for neuromorphic systems that encounter difficulties with nonlocal gradient transport.
- Scaling on analog substrates through partitioned emulation (Arnold et al.): analog and mixed-signal neuromorphic systems offer considerable advantages in energy efficiency and latency; however, they are constrained by the resources available on each chip. Arnold et al. introduced a partitioned emulation mode for BrainScaleS-2, enabling the virtualization of the substrate. This innovation allows larger neural networks to be divided into smaller subnetworks that can be executed sequentially, provided that the largest recurrent block fits within a single chip. They successfully demonstrated training models such as MNIST and EuroSAT that exceed the capacity of a single ASIC footprint.
- Many-core mapping that balances memory and MACs: Wang et al. demonstrate that simply having a high number of cores does not ensure high throughput on digital neuromorphic processors; careful mapping and scheduling are crucial for performance. They propose a spatial-temporal density mapping approach that considers both the location of data and the timing of computations. Two key factors contribute to performance improvements: better memory management to prevent fragmentation across cores and a scheduler that keeps the multiply-accumulate units active with minimal delays. On a many-core platform, this approach results in a significant system-level speedup compared to traditional layer-wise mapping, as shown through experiments on a large vision model.
- FPGA accelerator for deep SCNNs and residual topologies (Wu, Jiadong, et al.): The final article focuses on the efficient deployment of deep spiking convolutional neural networks (SCNNs) on field-programmable gate arrays (FPGAs). Wu, Jiadong, et al. designed a clock-driven accelerator that supports both standard and residual spiking neural network (SNN) architectures. Two key scheduling strategies are emphasized. The first strategy, called grouped reuse, involves rearranging computations so that groups of weights and feature maps are reused while they remain in on-chip memory. This approach helps to reduce the expensive off-chip memory traffic. The second strategy, known as line-by-line multi-timestep processing, allows several SNN timesteps to be processed for one spatial line of the feature map before moving on to the next line. This technique further enhances both reuse and pipeline efficiency. Together with channel-parallel pipelining, these strategies enable deeper SNNs to achieve high throughput while consuming less energy.

## 3 Key takeaways

The efficiency of computational processes can be optimized by improving model training, aligning hardware, and managing resources effectively:

- Efficiency is now often measured by the number of timesteps saved across various processes. Techniques that allow early inference termination or train models to perform well at shorter timesteps convert spike sparsity into significant savings in both time and energy without requiring changes to the model's architecture.
- Training must align with the hardware's communication model. Learning algorithms that avoid expensive backward passes while still providing reliable credit assignment are therefore a better fit for such hardware
- The performance of a system depends on effective mapping and scheduling, not just the number of cores. Improvements can be achieved through virtualization to address resource limitations or through spatial-temporal density mapping and specialized schedulers on many-core chips.

## 4 Open questions and next steps

- Benchmarking end-to-end energy: many studies suggest strong proxy metrics, including reduced timesteps, integer arithmetic, pipelining efficiency, and frames per second (FPS) at consistent clock speeds. Nevertheless, it remains challenging to compare neuromorphic techniques fairly.
- On-device adaptive computation: cutoff-aware training, dynamic thresholds, and gradient-free online updates suggest a future where devices routinely adapt latency and power to input difficulty and context. Nevertheless, there is unexplored potential in the close integration of event sensors (such as vision and audio) with closed-loop tasks.
- Mapping for emerging model families: as SNNs absorb ideas from modern deep learning (residual blocks, attention, spatio-temporal operations), compilers and runtimes must expose mapping knobs (partitioning, memory reuse, Psums bit-width management) that can be learned or auto-tuned per chip. Results here point to tangible wins from principled scheduling on both analog and digital platforms.
- Closer integration of neuroscientific model abstractions with hardware: Finally, there's increasing value in revisiting neuroscience-driven elements for neuromorphic substrates alongside deep-learning methods. Recent studies highlight advantages in modeling dendritic computation, synaptic delays, and local adaptive mechanisms, which are often either low-cost or inherent to neuromorphic hardware. These biologically inspired features are underutilized in mainstream spiking neural network (SNN) design, and exploring their potential could lead to significant efficiency and capability improvements.

